# Development and psychometric evaluation of Sexual and Reproductive Health Assessment Scale for women with educable intellectual disability (SRH-WIDS): a sequential exploratory mixed-method study

**DOI:** 10.1186/s12905-022-01755-5

**Published:** 2022-05-14

**Authors:** Abbas Ebadi, Ziba Taghizadeh, Maryam Farmahini Farahani

**Affiliations:** 1 Behavioral Sciences Research Center, Life Style Institute, Faculty of Nursing, Baqiyatallah University of Medical Sciences, Tehran, Iran; 2 Reproductive Health and Midwifery Department, Nursing and Midwifery Care Research Center, School of Nursing and Midwifery, Tehran University of Medical Sciences, Tehran, Iran; 3 Department of Midwifery, Faculty of Nursing and Midwifery, Tehran Medical Science, Islamic Azad University, Tehran, Iran

**Keywords:** Intellectual disability, Sexual and reproductive health, Scale

## Abstract

**Background:**

Women with Intellectual disability have many problems in their sexual and reproductive health due to their special mental and physical conditions caused by disability. This study aimed to develop and evaluate the psychometric properties of Sexual and Reproductive Health Scale for Women with Educable Intellectual disability.

**Methods:**

This sequential exploratory mixed-method study was conducted at two qualitative and quantitative stages in Tehran, from 2018 to 2020. At the qualitative stage, in-depth and semi-structured interviews were conducted with 21 parents and caregivers of women with intellectual disability to explain the concepts and to generate items. Thereafter, the item pool was formed based on the concepts and themes extracted from the qualitative phase as well as the review of literature. At the second stage, psychometric properties of the scale were assessed. Finally, responsiveness, interpretability, and feasibility of the scale were determined.

**Results:**

An item pool containing 95 items was produced at the first stage. At the second stage, the final version of scale was developed. The psychometric properties of this final version were then assessed and the results showed that the instrument has good validity and reliability. The results of exploratory factory analysis showed that the instrument contains seven factors. Accordingly, these factors explained 53% of the total variance of the instrument variables.

**Conclusions:**

The results show that the SRH-WIDS with 25 items has sufficient validity and reliability, so it could be easily used by caregivers to assess the sexual and reproductive health of Women with Educable Intellectual disability.

*Ethical code*: IRI.TUMS.VCR.REC 1397.340.

## Background

Sexual and reproductive health is considered as basic aspects of health, which complete the life of a human being. Notably, people with disabilities, as part of human diversities, have the same sexual and reproductive rights as other members of society [1].

Intellectual disability is a disorder beginning during the developmental period, which involves both simultaneous deficits in mental and adaptive performances in conceptual, social, and practical contexts [2].

Women with Intellectual disability have many sexual and reproductive problems due to their mental disabilities. According to previous studies, women with neurodevelopmental deficits experience pubertal changes earlier than their peers. Moreover, the lack of attention by parents and health care providers paid to this issue, as well as the lack of awareness of these women about puberty and related physical changes, can consequently cause great anxiety and emotional reactions in them [3]. Elikins et al. in their study showed that, due to the inability of women with Intellectual disability in expressing their emotions, the premenstrual symptoms would appear as the increased autistic behaviors, agitation, irritability, and seizures, which are more common among women with high degrees of intellectual disability [4]

Most women with intellectual disability are treated by antiepileptic drugs, which could lead to hormonal disorders, the decreased ovarian function, menstrual disorders and irregular bleeding due to their contrast with body's metabolic and hormonal functions [5].

Women with Intellectual disability are known as potential victims of sexual abuse. Their inability in understanding and expressing what has happened, their care for personal hygiene, and their lack of awareness about sexual relationships put them at the great risk of sexual abuse. Meanwhile, women with mild intellectual disability are at a greater risk due to their naivety, obedience, more presence in society, and most importantly, lack of knowledge and awareness of sexual issues [6].

The prevalence of sexual abuse in women with intellectual disability has been reported to be between 4 and 40% [7]. Notably, it is not possible to estimate the prevalence of sexual abuse accurately in them due to their inability to report sexual abuse, poverty, and sometimes their economic dependence on offender, as well as the lack of follow-up on sexual abuse [8]. Sexual abuse of women with intellectual disability besides the destructive and long-lasting effects on their souls and minds, increases the risk of infection by sexually transmitted diseases and AIDS.

Challenges in the sexual and reproductive life of women with intellectual disability are not necessarily due to their disability, but it can be a reflection of lacks of legal and social attention and support [9].

In 2015, World Health Organization specifically referred to the sexual and reproductive rights of people with intellectual disability, by stating that they have the right to receive sexual and reproductive health services, and sexual health information and education, and also they should not be subjected to coercive or involuntary procedures [10].

It is important to note that considering reproductive and sexual rights does not necessarily guarantee their implementations in everyday life [11]. Unfortunately, most people with intellectual disabilities not only have inadequate knowledge on their sexual and reproductive rights, but also are not supported to receive these rights [12]. Lacks of privacy, support for having a sexual partner, and support for marriage and parenting are some of the violations of these rights for them [13] Accordingly, women with intellectual disability not only need sexual and reproductive health services same as other women, but also because of their special conditions, this need is more felt in them compared to others. Moreover, among various degrees of intellectual disability, Women with Educable Intellectual disability face more social problems and challenges in sexual and reproductive health, may be due to the reason that this group of intellectual disabled has the highest percentage (75–90%) of the intellectual disabled population [14]. In addition, the possibility of their presence in society and their participation in social activities is higher than the groups with lower IQ. On the other hand, women with educable intellectual disability have a higher capacity for learning, so this potential can be used to promote their sexual and reproductive health.

It is necessary to have a specific, valid, and reliable instrument to study the sexual and reproductive health status of women with educable intellectual disability and to assess the related health service programs. A review of studies showed that although the World Health Organization has defined the domains of sexual and reproductive health, but there is no standard tool in this area and The available tools are specifically designed for different women such as conflict-affected women, women with specific diseases, women with breast cancer, and so on [15, 16]. Also, the results of studies have shown that one of the barriers against sexual health provision for people with intellectual disability is the lack of a valid Sexual and Reproductive Health Assessment Scale for this group [17, 18].

Few studies have been conducted on the sexual and reproductive health of women with intellectual disability in Iran and there are limited information in this area. Furthermore, in most studies, researcher-made questionnaires have been used that lacked psychometric properties and only limited aspects of sexual and reproductive health are addressed in them. Therefore, in this study, we attempted to design a sexual and reproductive health instrument for women with educable intellectual disability based on the perspective of their caregivers in a qualitative approach as well as evaluating its psychometric properties in a quantitative approach with the hope of taking a step to improve sexual and reproductive health of this group of women community.

## Materials and methods

This was a Sequential Exploratory Mixed Method study conducted in two phases in Tehran between 2018 and 2020. The first phase of the study was performed using a qualitative approach to produce items and design the instrument and in the second phase, the psychometric properties of the instrument were examined.

### Phase 1: Production of items and design of instrument

#### Research design

In this research, the inductive-deductive approach was used to produce instrument’s items. One of the advantages of using this approach is obtaining direct information from the study’s participants and then using the available literature and instruments in this field, which would cover the subject from all aspects.

#### Participants and data collection

In the first step, the qualitative study was performed to explain caregivers’opinions regarding sexual and reproductive health of women with intellectual disability. For this purpose, we selected the participants using targeted sampling, with maximum variation possible in terms of age, degree of education, and work experience to obtain different opinions about the phenomenon from the best informants [19]. Therefore, in-depth and semi-structured interviews were conducted by including 21 participants who were parents, caregivers, and health professionals of women with intellectual disability in care centers and daily rehabilitation centers in Tehran. Health professionals participating in this study included gynecologist, midwife, psychologist, General practitioner, teacher, professional instructor and Executive Director of intellectual Disabled care centers. The age range of interviewed participants was between 28 and 62 years old and the range of work experience of caregivers was 3–26 years. The interviews were conducted face to face and individually and were recorded by a tape recorder. Interviews were transcribed by the researcher immediately after recording.

Based on the extracted concepts in proportion to the Iranian cultural and social contexts, a major part of the instrument’s items was provided. Thereafter, by reviewing literature about sexual and reproductive health of women with intellectual disability, another part of the items was extracted and the initial item pool was then formed in the deductive method.

#### Data analysis

In this study, qualitative data were analyzed by conventional content analysis method proposed by Wildemuth (2016) using MAXQDA10 software [20]. Afterward, based on the extracted concepts, 83 instrument’s items were provided. Next, a deductive approach was used to complete the items and 12 items were then adapted from the review of studies and questionnaires in this field. Finally, an item pool consisting of 95 items was formed. After reviewing the obtained items by members of the research team, numbers of the items were removed or merged, and the initial version of the instrument was designed with 43 items.

### Phase 2: Psychometric evaluation of the instrument

#### Research design

Psychometric properties of the Sexual and Reproductive Health Assessment Scale for Women with Educable Intellectual disability (SRH-WIDS) were evaluated in a quantitative approach.

#### Sampling

In the present study, exploratory factor analysis was used to determine the construct validity. It is noteworthy that the sample size in factor analysis depends on the number of instrument items, and for this study, it was recommended to be at least 3–10 participants for each item [21]. Thus, 202 family caregivers of educable intellectual disabled women who responsible for caring of them in the age range of 15–45 years old (Reproductive age), were selected through convenient sampling. All daily rehabilitation centers for women with intellectual disability in Tehran were selected as the research environment and family caregivers who referred to these centers, were invited to participate in this study.

#### Statistical analysis

In quantities phase, the obtained data were analyzed using several statistical methods in SPSS 23 software to determine the psychometric properties of the instrument:

1. Validity: We examined the face, content, and construct validities of the instrument.

### Face validity

Quantitative and qualitative methods were used to determine the face validity of the instrument. In the qualitative method, the difficulty of understanding, the possibility of misunderstandings, and inadequacy in the meaning of the items were examined by 10 family caregivers of educable intellectual disabled women, and necessary corrections were then made. Thereafter, in order to evaluate the face validity in a quantitative method, the item impact method was used. In this method, for each one of the instrument’s items, a five-point Likert scale was considered (quite important = 5, somewhat important = 4, moderately important = 3, slightly important = 2, and not important at all = 1). Subsequently, ten caregivers were asked to determine the importance of each item based on their own experiences. The impact score of each item was then calculated based on the following formula. In this formula, frequency refers to the percentage of the participants who gave each item scores of 4 and 5, and importance refers to the mean scores of importance based on the Likert scale.

$${\text{Impact Score}} = {\text{Frequency}}\;\left( \% \right)*{\text{Importance}}$$

Items with an impact score greater than 1.5 were considered as appropriate ones [22].

### Content validity

A team consisted of ten researchers specialized in psychometric tool, sexual and reproductive health, and exceptional children psychology examined the content validity of the instrument, both qualitatively and quantitatively. At the qualitative stage, sentence structure, grammar, identifying and placing the items in their proper places were evaluated. At the quantitative step, content validity ratio (CVR), content validity index (CVI), and scale-level content validity index/averaging calculation method (S-CVI / Ave) were calculated [22].

Notably, content validity ratio examines the item essentiality from the perspective of experts. While in the content validity index, the relevance of the items to the purpose of the research is considered by experts [23].

Acceptable values of CVR, I-CVI, and S-CVI/Ave were considered as equal to and more than 0.62, equal to and more than 0.78, and equal to and more than 0.90, respectively [24, 25].

### Pilot study

Performing a preliminary study with them aim of assessing the initial reliability helps to identify possible problems in the implementation phase [26]. In this study, the internal consistency method was examined by calculating Cronbach's alpha coefficient for each item (above 0.7 is the desirability criterion), as well as item-total correlation (above 0.3 is the desirability criterion) in a sample of 30 Caregivers [27].

### Construct validity

Factor analysis is considered as one of the best methods for determining the construct validity [28]. Therefore, in the present study, the exploratory factor analysis method was used to determine the construct validity. Furthermore, the five-step guide proposed by Williams et al. (2010) was used to conduct exploratory factor analysis [29]. Accordingly, The Kaiser-Meyer Olkin (KMO) and Bartlett's Test measure of sampling adequacy were used to examine the appropriateness of Factor Analysis. In this regard, Maximum likelihood and Varimax rotation methods were used to extract the factors and several criteria such as Kaiser’s criterion (eigenvalue more than one), scree plot, and cumulative variance explained by all the extracted factors were also examined. Additionally, in this study, the minimum acceptable factor load for the items was considered to be at least 0.3. Finally, the extracted factors were named [30].

### Reliability

In this study, the internal reliability and the external reliability of the instrument were calculated by measuring the internal consistency, and Inter Class Correlation (ICC) and Standard Error of Measurement (SEM), respectively. Internal consistency refers to the correlation between the items of instrument. Cronbach's alpha was then calculated to measure internal consistency. Values equal to or greater than 0.60 were considered satisfactory [31].

To calculate ICC, the designed instrument was completed by 30 participants, and then re-completed by the same people after two weeks. A correlation coefficient 1- 0.8 indicates excellent reliability [32].

### Responsiveness

The responsiveness or sensitivity of an instrument indicates its ability to detect changes over time. In this study, the responsiveness of the instrument was determined by examining the measurement error using standard error of measurement (SEM) and minimum detectable change (MDC) methods [33, 34]. MDC 30% is acceptable and less than 10% is considered as excellent [35].

### Interpretability

Interpretability is the degree of ability to refer qualitative meanings to quantitative scores. In this study, based on the COSMIN checklist, the criteria that fall into the field of interpretability were as follows: determining the percentage of the missing items, adequacy of the sample size, describing the distribution of total scores in the samples, determining the ceiling and floor effect, and evaluating Minimal Important Changes (MIC) [36].

To calculate MIC, the standard deviation of the changes between test–retest can be multiplied by the average effect size, which is 0.5 in this study [37]. Notably, the MIC must be larger than the MDC [23]

Another indicator of the interpretability is the determination of the ceiling and the floor effects. Correspondingly, the ceiling effect occurs when most respondents choose the upper limit of a scale, and the floor effect occurs when most participants select the lower limit of the scale [38]. In addition, this index should be less than 15% [39].

Another method of confirming interpretability is examining the distribution of scores in the samples and the mean and standard deviation of the variables are expected to be vary in different groups. Accordingly, the mean score of sexual and reproductive health in different groups of participants was calculated based on the designed instrument. Moreover, another method of verifying interpretability is calculating the percentage of the missing items. Accordingly, if this value is between 15 and 20%, it is desirable [40]

### Ethics

For performing this study, an approval was taken from the ethics committee of Tehran University of Medical Sciences, Iran (IRI.TUMS.VCR.REC 1397.340). All the participants were also informed that participation in this study would be voluntary and that the confidentiality of their information would be maintained. In addition, informed written consent was obtained from all the included participants.

## Results

### Participants

202 family caregivers of educable intellectual disabled women who responsible for caring of them in the age range of 15–45 years old (reproductive age), were selected through convenient sampling. The minimum age of the participants was 32 years old and the maximum was 84 years old (mean age, 56.4). Their levels of education ranged from illiterate to university degrees and their economic statuses ranged from good to poor. The characteristics of the study’s participants are shown in Table 1.

**Table 1:** Participants’ demographic characteristics

Characteristics	Categories	Frequency	Percent
Age (years)	30–45	25	12/4
	45–60	120	59/4
	60–75	51	25/2
	75–90	6	3
Educational status	Illiterate	28	13/9
	High school	74	36/6
	Diploma	74	36/6
	Academic	26	12/9
Economic statuses	Good	13	6/4
	Moderate	149	73/8
	Poor	40	19/8

### Validity

#### Face validity

At the qualitative stage of face validity, 4 items were modified due to the difficulty in understanding their meanings for the participants. At the quantitative stage of face validity, all the items had impact scores above 1.5; therefore, no item was deleted.

#### Content validity

In the qualitative phase of content validity, all the changes proposed by the experts were made on the items. At this stage, the writing style of eleven items was corrected and two items were merged. In a quantitative phase of content validity, 7 items with a CVR less than 0.62 and 4 items with a CVI less than 0.78 were omitted. The S-CVI of the instrument was also calculated as 0.915. Finally, the instrument was prepared with 31 items to enter the preliminary study stage.

### Pilot study

At this stage, two items were omitted due to item-total correlation less than 0.3. Moreover, internal consistency was examined with the remaining 29 items, and the Cronbach's alpha coefficient of the total instrument was calculated as 0.80.

#### Construct validity

Exploratory factor analysis was used to evaluate the construct validity. Kaiser–Meyer–Olkin (KMO) and Bartlett tests showed that the data were suitable for factor analysis. Accordingly, the results of the KMO and Bartlett tests are shown in Table 2.

**Table 2:** KMO sampling adequacy index and Bartlett test results

KMO and Bartlett's test		
Kaiser–Meyer–Olkin measure of sampling adequacy		0.758
Bartlett's test of sphericity	Approx. Chi-square	1920.123
	*df*	300
	Sig	*P* < 0.001

The Maximum Likelihood method and Varimax orthogonal rotation under the assumption of independence for the factors were selected for the initial extraction of latent factors.

**Fig. 1:**
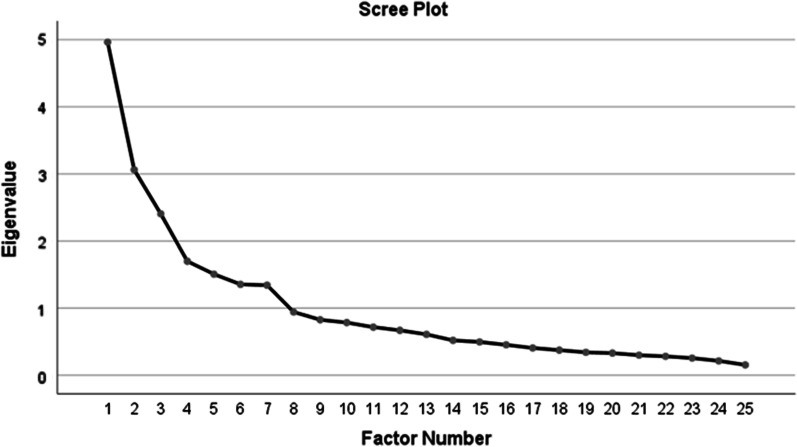
Scree plot

Finally, according to the results obtained from the scree plot (Fig. 1) and based on the opinion of the research team, the seven-factor model with eigenvalues higher than 1 and factor loading equal to or greater than 0.3 was confirmed. At this stage, 4 items were omitted due to having factor loads of less than 0.3. In total, the factors explained 53% of the total variance of the instrument variables.

The first factor with 4 items was named as “independence in personal and menstrual hygiene”, the second factor with 6 items as “inappropriate sexual behavior” the third factor with 3 items as “sexual self-care education”, the fourth factor with 3 items as “Privacy recognition”, the fifth factor with 3 items as “sexual and reproductive knowledge”, the sixth factor with 4 items as “control of emotions”, and the seventh factor with 2 items was named as “menstrual concerns”. Each factor is shown in Table 3 based on the relevant items and in order of factor loading.

**Table 3:** Labeling each factor based on the relevant items by factor loading

Items	Factor loading						
	Factor 1	Factor 2	Factor 3	Factor 4	Factor 5	Factor 6	Factor 7
She has the ability to have menstrual hygiene	0.906						
She is dependent on me to change the sanitary pad	0.872						
She has the ability to have personal hygiene	0.614						
She needs help to carry out the activities of daily living	0.570						
When she sees sexual scenes, he imitates their content in the presence of others		0.721					
She has sex with his homosexual		0.720					
She behaves inappropriately towards the opposite sex (hugging, kissing, touching, showing her genitals)		0.654					
She has been sexually abused		0.638					
She talks to others about her sexual fantasies		0.612					
She masturbates in public places		0.358					
She is taught how to take care of herself against sexual abuse			0.820				
She is taught about privacy			0.746				
She is taught about sexual relationship			0.509				
She knows the private parts of the body				0.808			
She knows privacy				0.788			
She distinguishes the stranger				0.495			
She knows contraceptives methods					0.867		
She knows the possible consequences of having sex					0.684		
She has a correct knowledge of marriage issues					0.539		
She understands the emotional changes of others (happiness, sadness, fear and anger)						0.593	
She behaves appropriately to express her feelings to others: such as greeting, shaking hands, kissing and hugging						0.584	
She does not like to communicate with others						0.499	
She is anxious to be in public						0.452	
She becomes aggressive before menstruation							0.771
Her menstrual pains are unbearable							0.701

### Reliability

Cronbach's alpha coefficient of the total instrument was obtained as 0.80. In addition, Inter Class Correlation (ICC) of the total instrument was obtained 0.97. The Standard Error of Measurement (SEM) of the total instrument was also desirable. Altogether, these values indicate the optimal reliability of the instrument. Besides, all these values were separately calculated for each factor. Two factors had lower alpha coefficient than the others. Because alpha coefficient may not be an optimal estimator of internal consistency reliability, when the number of items is small, the other two measures of scale should be interested (theta and omega) and the omega coefficient is based on factor analysis [41], so we decided to measure omega coefficient. Fortunately, the results of the omega reliability study were satisfactory. All these values are shown in Table 4

**Table 4:** The Reliability of sexual and reproductive health scale for women with educable intellectual disability

No	Factor	Mean	SD	α	Omega	ICC	CI = %95	SEM	Result
1	Independence in personal and menstrual hygiene	9.6	7.3	0.857	0.858	0.978	0.990–0.954	0.54	Excellent
2	Inappropriate sexual behavior	22.85	2.6	0.780	0/741	0.998	0.999–0.995	0.11	Excellent
3	Sexual self-care education	4.7	2.04	0.762	0.815	0.863	0.935–0.713	0.08	Excellent
4	privacy recognition	8.2	2.65	0.803	0.858	0.970	0.986–0.937	0.45	Excellent
5	Sexual and reproductive knowledge	1.06	1.96	0.678	0.832	0.967	0.984–0.930	0.3	Satisfactory
6	Control of emotions	11.75	2.95	0.613	0.710	0.974	0.988–0.946	0.5	Satisfactory
7	Menstrual concerns	5.8	1.97	0.717	0.827	0.960	0.981–0.916	0.39	Excellent
Total		64.24	10.3	0.809	0.796	0.976	0.988–0.949	1.5	Excellent

SD: Standard deviation; ICC: interclass correlation coefficient; CI: confidence interval; SEM: standard error of measurement

### Responsiveness

The MDC% was obtained as less than 30% for each factor and 6.46% for the total instrument. Furthermore, the amount of SEM obtained for each factor and the total instrument indicates the desirable Responsiveness of the instrument.

### Interpretability

The criteria of Interpretability were determined based on the COSMIN checklist. By calculating the MIC and its larger value than MDC (5.15 > 4.15), it was found that the designed instrument is able to detect Minimal Important Changes in measuring sexual and reproductive health levels, so reporting changes in scores is not due to measurement error and it is reliable. The ceiling and floor effects were separately calculated for each factor. Both of the ceiling and the floor effects were separately less than 15% for the total instrument and its factors, which are acceptable. Besides, by examining the distribution of scores in the samples, it was found that the mean scores of sexual and reproductive health are statistically significantly different among various participants’ educational classes and age groups. Finally, by calculating the percentage of the missed items, it was found that 99.2% of the items were answered by the participants and only 0.8% of the items were missed.

### Scoring

In this study, the five-point Likert scale was used for scoring the instrument. The score of 12 items were counted inversely. To better understand the scoring process and their comparability, the scores of each factor were converted to scores from zero to one hundred, and scoring this instrument was also considered from zero to one hundred. This conversion was performed for each factor using the linear conversion scoring method. The closer an individual’s score was to 100, the better her sexual-reproductive health level.

$$\begin{aligned} &{\text{Score of each factor}}\\ &\quad= \frac{{{\text{least possible score}} - {\text{the score obtianed in the factor}}}}{{{\text{least possible score}} - {\text{maximum possible score}} }}\\ &\qquad\times 100\end{aligned}$$

## Discussion

This study provided a sexual and reproductive health scale for women with educable intellectual disability (SRH-WIDS). The SRH-WIDS contains 25 items incorporated into 7 factors. Correspondingly, the first factor consists of 4 items with a factor load between 0.57 and 0.90, which has the highest percentage of total variance (10.5%). Notably, it was named as “Independence in personal and menstruation hygiene.”

This dimension of the instrument evaluates the ability of women with educable intellectual disability for managing menstruation. Based on their degrees of intellectual disability, women with intellectual disability depend on their caregivers for their menstrual health and personal hygiene. Dependency on others for self-care would have a destructive impact on their feminine identity. Ditchfield [42] in his qualitative study, pointed out that the ability of women with educable intellectual disability in managing their menstruation can strengthen their feminine identity.

The second factor of the instrument was named as “inappropriate sexual behavior” with 6 items and a factor load between 0.35 and 0.72. This dimension of instrument accounted for 10.05% of the total variance. Its items are about the occurrence of inappropriate sexual behaviors such as inappropriate social behaviors with the opposite sex, masturbation in public places, exhibitionism, inappropriate touching of others, and sexual abuse.

Ward et al. (2001) reported that the most common type of inappropriate sexual behavior in people with developmental disabilities is sexual behaviors in public situations. These behaviors include public exposure, public masturbation, public stripping, and consensual sexual behavior in public [43].

The third factor of the instrument was called “sexual self-care education” with 6 items and a factor load between 0.50 and 0.82. This dimension of the instrument accounts for 7.68% of the total variance. Additionally, the fourth factor was called “privacy recognition” with 3 items and a factor load between 0.49 and 0.80. This dimension of the instrument accounts for 7.22% of the total variance.

Self-care training in sexual abuse prevention includes privacy recognition; understanding different parts of the body such as sexual and private organs; distinguishing family members, friends, family, and strangers; recognizing sexual abuse; and how to get help [44]. Monaco et al. [45] considered that the training of self-confidence, self-control, and autonomy as the sexual teachings and also considered the training of these topics as necessary for achieving sexual and reproductive health of women with intellectual disabilities.

The fifth factor of the instrument was called “sexual and reproductive knowledge” with 3 items and a factor load between 0.53 and 0.86, which accounts for 6.70% of the total variance.

In 2015, World Health Organization specifically referred to the sexual and reproductive rights of people with intellectual disabilities and stated that they had the right to receive information and education about their sexual health [13]. However, in fact, the sexual knowledge of people with intellectual disabilities is very little, so providing information about sexual issues is necessary for this group [46].

The sixth factor of the instrument was called “control of emotions” with 4 items and a factor load between 0.42 and 0.59. This dimension of the instrument accounts for 5.93% of the total variance. The items related to this factor are about how to express feelings to others.

People with intellectual disability may have difficulty in understanding boundaries in social levels of intelligence [44]. Emotional stability is strongly linked with sexuality and failure in understanding and managing emotions, which cause communication limitations and failure in love relationships [45]. The seventh factor of the instrument was called “menstrual concerns” with 2 items and a factor load between 0.70 and 0.77. This dimension of the instrument accounts for 4.90% of the total variance. Although this factor has two items, according to Yong and Pearce [47], because their specific factor load is more than 0.7, it is also considered as a factor. The items related to this factor are related to premenstrual symptoms such as mood swings and menstrual cramps. Elikins et al. [4] in his study reported that in 32% of women with intellectual disability, premenstrual symptoms are characterized by the increased autistic behaviors, irritability, insomnia, and seizures. The occurrence of these symptoms is more among those women with intellectual disability who have communication problems and are not able to express their feelings and to report their symptoms [48].

The results of this study show that the SRH-WIDS have desirable psychometric properties. The strength of this instrument is that, it has been tested using various validity and reliability methods. Moreover, responsiveness and interpretability of the instrument have been determined and all these tests have been desirable. Also, examining the two criteria, i.e. instrument response time (average 15 min) and the percentage of missing items (0.8%), showed that feasibility of scale is desirable. This instrument can be used to assess the sexual and reproductive health status of Women with Educable Intellectual Disability. Since the Sexual and reproductive health status of women with intellectual disability is a reflection of the performance of their caregivers in this area, the results of such assessment can be a good guide to improve current approaches and interventions in this area.

## Limitations

The present study also had some limitations. Some parents were reluctant to participate in the study due to the sensitivity of the topic and the shame of talking about sexual issues, so the researcher tried to explain the importance of research, establish a friendly interaction with participants, and spend enough time in the presentation and observance of ethical principles in order to gain the trust and confidence of the participants for reducing this limitation. Another limitation of the study was the lack of access and interviewing with family caregivers who were keeping educable intellectual disabled women at home and did not bring them to rehabilitation and training centers. Because sexuality is a very private subject with varying degrees of social, cultural, religious, moral, and legal norms and constraints [49], it is recommended to examine this instrument in different cultures and societies. Also, due to time constraints, confirmatory factor analysis was not performed, so it is recommended to perform confirmatory factor analysis for the purpose of confirming the construct validity of the instrument in future research.

## Conclusions

SRH-WIDS was designed based on inductive-deductive approach to evaluate the sexual and reproductive health statuses of women with educable intellectual disability. Accordingly, it has sufficient validity and reliability, so it could be easily used by their caregivers. The basic information about the current state of sexual and reproductive health of women with educable intellectual disability could be provided using this instrument. This information can be considered as a good guidance for health care providers of women with intellectual disability to review the current laws and policies in the field of sexual reproductive health, so that they can finally design new strategies and approaches to promote their sexual reproductive health.

## Data Availability

The datasets supporting the conclusions of this article are included within the article.

## Abbreviations

SRH-WIDS: Sexual and Reproductive Health Assessment Scale for women with educable intellectual disability
CVR: Content validity ratio
CVI: Content validity index
S-CVI/Ave: Scale-level content validity index/averaging calculation method
KMO: Kaiser–Meyer Olkin
ICC: Inter class correlation
SEM: Standard error of measurement
MDC: Minimum detectable change
MIC: Minimal important changes
